# Trends and Disparities in Treatment for Co-occurring Major Depression and Substance Use Disorders Among US Adolescents From 2011 to 2019

**DOI:** 10.1001/jamanetworkopen.2021.30280

**Published:** 2021-10-20

**Authors:** Wenhua Lu, Miguel Muñoz-Laboy, Nancy Sohler, Renee D. Goodwin

**Affiliations:** 1 Department of Community Health and Social Medicine, School of Medicine, The City University of New York, New York; 2 School of Social Welfare, Stony Brook University, New York, New York; 3 Department of Epidemiology and Biostatistics, Graduate School of Public Health and Health Policy, The City University of New York; 4 Department of Epidemiology, Mailman School of Public Health, Columbia University, New York, New York

## Abstract

**Importance:**

Major depression and substance use disorders (SUD) commonly co-occur among adolescents, yet little is known about treatment use among adolescents with both conditions. Given the reciprocal influence of these conditions on each other and low prevalence of treatment overall, current information on quantification and trends in treatment of co-occurring depression and SUD is critical toward assessing how the field is performing in reaching youth in need of these services, and among youth with sociodemographic risk factors.

**Objective:**

To examine temporal trends and sociodemographic disparities in the treatment of co-occurring major depression and SUD among US adolescents.

**Design, Setting, and Participants:**

This survey study used publicly available data for adolescents aged 12 to 17 years from the annual cross-sectional surveys of the National Survey on Drug Use and Health from 2011 to 2019 to assess co-occurrence of major depressive episodes (MDE) and SUD through time and prevalence of treatment for either or both of these conditions. Data were analyzed between October 2020 and February 2021.

**Exposures:**

Survey years, adolescent age, gender, race and ethnicity, type of insurance, annual household income, family structure, and residential stability.

**Main Outcomes and Measures:**

Presence and treatment of co-occurring 12-month MDE and SUD.

**Results:**

In total, 136 262 adolescents participated in the 2011 to 2019 surveys, among whom 69 584 (51.1%) were boys and 66 678 (49.0%) were girls, 46 548 (34.1%) were aged 16 to 17 years, and 18 173 (13.8%) were Black, 28 687 (23.2%) were Hispanic, and 74 512 (53.6%) were White. From 2011 to 2019, the annual prevalence of co-occurring MDE and SUD remained stable, at between 1.4% and 1.7%. Among adolescents with co-occurring MDE and SUD, the prevalence of treatment use for MDE only increased significantly from 28.5% in 2011 to 42.5% in 2019 (odds ratio [OR], 1.07; 95% CI, 1.02-1.11; *P* = .005), whereas the prevalence of treatment use for SUD only decreased from 4.8% to 1.5% (OR, 0.92; 95% CI, 0.85-0.99; *P* = .04). Overall, the prevalence of treatment use for both conditions fluctuated between 4.5% and 11.6%, without a significant linear trend over time (OR, 0.95; 95% CI, 0.87-1.03; *P* = .24). Extensive disparities in treatment use were found among boys for SUD and both conditions, older adolescents for MDE, Hispanic adolescents for co-occurring conditions (adjusted OR, 0.52; 95% CI, 0.27-0.98; *P* = .04), and Asian, Native Hawaiian, or Pacific Islander adolescents for MDE (adjusted OR, 0.24; 95% CI, 0.10-0.58; *P* = .002) and co-occurring conditions (adjusted OR, 0.04; 95% CI, 0.01-0.33; *P* = .003). Moving households 3 or more times in the past 12 months was associated with higher odds that adolescents received treatment for both conditions (adjusted OR, 2.52; 95% CI, 1.26-5.05; *P* = .009).

**Conclusions and Relevance:**

This survey study found that from 2011 to 2019, less than 12% of adolescents with major depression and SUD received treatment for both conditions from 2011 to 2019. Findings from this study call for expanded service provision for adolescents with co-occurring conditions, improved coordination between service delivery systems, and enhanced policy and funding support for adolescents with unmet treatment needs.

## Key Points

**Question:**

What was the prevalence of treatment use for co-occurring major depression and substance use disorders among adolescents in the US from 2011 to 2019, and were there any disparities?

**Findings:**

This survey study including 136 262 adolescents aged 12 to 17 years found persistent treatment gaps for co-occurring major depression and substance use disorders. Unmet treatment needs were significantly higher among Hispanic and Asian, Native Hawaiian, or Pacific Islander adolescents and uninsured adolescents.

**Meaning:**

The findings of this study suggest that continued efforts to improve service provision and coordination for adolescents with co-occurring depression and SUD are needed.

## Teaser

This survey study examines temporal trends and sociodemographic disparities in the treatment of co-occurring major depression and substance use disorders among adolescents in the National Survey on Drug Use and Health from 2011 to 2019.

## Introduction

Major depression and substance use disorder (SUD) both first emerge most commonly during adolescence^1,2,3^ and are each associated with severe health and social consequences in late adolescence (eg, self-injuries, academic failure, violence, suicide) and adulthood (eg, sexual abuse, homelessness, unemployment, crime).^4,5^ Compared with adolescents with either depression or SUD alone, adolescents with both conditions are at higher risk for these negative consequences. Emerging research has documented an upward trend in adolescent depression and, in contrast, an overall downward trend in SUD in recent years,^6,7,8^ yet little is known about the trend of their co-occurrence in national samples. Given the severe consequences of depression and SUD, routine monitoring of their co-occurrence is critical to inform prevention and intervention.^9,10^

Theoretically, adolescents with co-occurring depression and SUD should receive treatment for both conditions.^9,11^ Research indicates that treating depression alone does not significantly reduce SUD,^12,13^ and SUD treatment alone does not result in remission of depression.^12^ Unfortunately, we lack comprehensive knowledge about trends and patterns in adolescents’ unmet treatment needs for co-occurring conditions. Furthermore, prior findings have identified disparities in adolescent depression and SUD based on certain demographic, family, and social characteristics (eg, gender, race, family structure, residential stability).^14^ Yet, relatively little is known about potential corresponding disparities in unmet treatment need for co-occurring conditions. This information has direct implications for improving implementation of evidence-based practices, coordinating service delivery, and expanding treatment access for underserved adolescents.

The purpose of this study was to address these research gaps using nationally representative data for adolescents ages 12 to 17 years from National Survey on Drug Use and Health (NSDUH). Specifically, this study aimed to examine temporal trends in the prevalence and treatment of co-occurring adolescent depression and SUD and identify disparities in unmet treatment needs among adolescents with both conditions.

## Methods

This survey study was approved by the institutional review board at RTI International. Verbal informed consent was obtained from a parent or guardian, and verbal assent was obtained from each adolescent participant before survey administration. This study followed the American Association for Public Opinion Research (AAPOR) reporting guideline for survey studies.

### Sample

The NSDUH is an annual cross-sectional survey sponsored by the Substance Abuse and Mental Health Services Administration within the US Department of Health and Human Services.^15^ Using a stratified multistage area probability sampling method, the NSDUH provides nationally representative data for the civilian, noninstitutionalized population aged 12 years or older from all 50 states and the District of Columbia.^15^ For this study, publicly available data for adolescents aged 12 to 17 years in 2011 to 2019 were analyzed. The survey is administered in English and Spanish, and interviews are conducted using computer-assisted interviewing.

### Measures

#### Major Depression

Major depression in the NSDUH was measured using a structured interview based on the *Diagnostic and Statistical Manual of Mental Disorders* (Fourth Edition) (*DSM-IV*).^16^ Adolescents were classified as having a 12-month major depressive episode (MDE) if they had experienced either depressed mood or loss of interest or pleasure in daily activities for 2 weeks or longer in the past 12 months, while also experiencing 4 or more other symptoms that reflect a change in functioning, such as problems with sleep, eating, energy, concentration, and self-worth.^17^

Adolescents with 12-month MDE were further asked questions from the Sheehan Disability Scale to measure the level of MDE-related functional impairment in 4 major life activities or role domains (ie, chores at home, school or work, close relationships with family, and social life). On a 0 to 10 visual analog scale with categories of no interference (0), mild (1-3), moderate (4-6), severe (7-9), and very severe (10), ratings of 7 or greater were considered severe impairment. For this study, we dichotomized participants as having MDE-related severe functioning impairment or not.^17^

#### Substance Use Disorders

In the NSDUH survey, adolescents were asked about their use of alcohol and illicit drugs (including marijuana, hallucinogens, inhalants, cocaine, or heroin, or nonmedical use of psychotherapeutics, such as tranquilizers, pain relievers, sedatives, or stimulants) in the past year. If respondents reported using a substance in the past year and on more than 5 days for alcohol and marijuana, they were further asked questions that correspond to the *DSM-IV* criteria for dependence or abuse (eg, tolerance, withdrawal, taking larger amounts or taking them for longer periods, inability to cut down, time spent using the substance, giving up activities, and continued use despite problems) in the past year.^16^ Adolescents were categorized as having 12-month SUD if they met *DSM-IV* criteria for an alcohol or illicit drug dependence or abuse.

#### Treatment

To assess treatment for depression overall, adolescents with 12-month MDE were asked if they had seen or talked to a physician or other professional about their MDE symptoms. To assess treatment for SUD overall, adolescents were asked if they had used treatment or counseling designed to help reduce or stop alcohol or drug use at any location in the past 12 months, including hospital (inpatient), rehabilitation facility (inpatient or outpatient), mental health center, emergency department, private physician’s office, prison or jail, or self-help group, such as Alcoholics Anonymous or Narcotics Anonymous. Adolescents were considered as receiving treatment for MDE and SUD if they responded yes to both questions.

All adolescents in the NSDUH were asked if they had received “any treatment or counseling for behavioral or emotional problems that were not caused by alcohol or drug use” from 11 sources of service, such as hospital (inpatient), day treatment program, mental health clinic, school-based mental health professionals, and pediatrician or family physician. Although these questions did not target adolescents with MDE specifically, they were used as proxy measures of sources of MDE treatment use in this study.

#### Sociodemographic Characteristics

Sociodemographic variables were self-reported and included adolescents’ age group (12-13, 14-15, and 16-17 years), gender (male, female), race and ethnicity (White, Hispanic, non-Hispanic Black, Asian or Native Hawaiian or other Pacific Islanders, or other), insurance type (uninsured, Medicaid or Children’s Health Insurance Plan, private insurance, or other insurance), annual household income (<$20 000 to ≥$75 000), family structure (whether they had a father or a mother in the household), and residential stability (times that the participant moved in the past 12 months). Race and ethnicity were included to represent adolescent social experiences. Family structure and residential instability were included because they are often considered markers associated with underlying social, educational, and familial disadvantage for adolescents.^18,19^

### Statistical Analysis

Bivariate logistic regression analyses were first conducted to assess trends in the prevalence and treatment of co-occurring MDE and SUD overall and by sources of service, with survey year as the continuous independent variable. Following that, a series of multivariate logistic regression models were used to identify sociodemographic differences in the prevalence and treatment of co-occurring MDE and SUD. To adjust for potential time influence, the survey year variable was included in all multivariate models. Adolescents’ MDE-related severe impairment was further added in the multivariate models for treatment to adjust for the potential influence of symptom severity; adjusted odds ratios (AORs) are reported. All analyses were performed using R statistical software version 4.0.3 (R Project for Statistical Computing), accounting for the complex survey design, nonresponse bias, and noncoverage bias by using sampling weights provided by the NSDUH. In the NSDUH surveys from 2011 to 2019, the weighted response rates for adolescents ranged from 70.5% to 85.0%.^15^ Missing data ranged from 0% to 2.7% for the variables included in this study. Given that missing data were minimal,^20^ we excluded participants with missing values, as recommended by the NSDUH.^15^ *P* values were 2-sided, and statistical significance was set at *P* = .05. Data were analyzed between October 2020 and February 2021.

## Results

In total, 136 262 adolescents participated in the NSDUH from 2011 to 2019, among whom 68 584 (51.1%) were boys and 66 678 (49.0%) were girls, 46 548 (34.1%) were aged 16 to 17 years, and 18 173 (13.8%) were Black, 28 687 (23.2%) were Hispanic, and 74 512 (53.6%) were White. A total of 78 885 adolescents (58.5%) had private insurance, and 50 947 adolescents (39.8%) had annual household income more than $75 000 (Table 1). While 37 968 adolescents (26.1%) did not have a father living in their household, 11 605 adolescents (8.3%) did not have a mother living in their household. Most adolescents (98 708 adolescents [74.5%]) had not moved households in the last 12 months. In total, 2072 adolescents (1.5%) had co-occurring MDE and SUD.

**Table 1.** Sociodemographic Characteristics of All Adolescents in the National Survey on Drug Use and Health From 2011 to 2019

Characteristic	Adolescents, No. (%) (N = 136 262)^a^
Gender	
Boys	69 584 (51.1)
Girls	66 678 (49.0)
Age, y	
12-13	43 547 (31.8)
14-15	46 167 (34.1)
16-17	46 548 (34.1)
Race and ethnicity	
Asian, Native Hawaiian, or Pacific Islander	5699 (5.6)
Black	18 173 (13.8)
Hispanic	28 687 (23.2)
White	74 512 (53.6)
Other^b^	9191 (3.8)
Insurance coverage	
None	7859 (6.6)
Medicaid or CHIP	46 094 (33.5)
Private insurance	78 885 (58.5)
Other insurance	2003 (1.4)
Household income, $	
<20 000	23 261 (16.4)
20 000-49 999	40 112 (28.6)
50 000-74 999	21 942 (15.2)
≥75 000	50 947 (39.8)
Father in household	
Yes	98 170 (73.9)
No	37 968 (26.1)
Mother in household	
Yes	124 542 (91.7)
No	11 605 (8.3)
Times family moved in past 12 mo	
None	98 708 (74.5)
1	19 627 (14.5)
2	9594 (7.4)
≥3	4956 (3.6)

Abbreviation: CHIP, Children’s Health Insurance Program.

^a^ Unweighted sample sizes and weighted percentages are reported.

^b^ Includes individuals identifying as more than 1 race or ethnicity or as none of the provided race and ethnicity options.

### Trends in the Prevalence and Treatment of Co-occurring MDE and SUD

From 2011 to 2019, there was no statistically significant linear trend in the annual prevalence of co-occurring MDE and SUD, with 1.5% (280 of 19 264 adolescents) in 2011, 1.5% (280 of 17 399 adolescents) in 2012, 1.4% (259 of 17 736 adolescents) in 2013, 1.4% (188 of 13 600 adolescents), in 2014 1.4% (205 of 13 585 adolescents) in 2015, 1.3% (192 of 14 272 adolescents) in 2016, 1.4% (213 of 13 722) in 2017, 1.5% (214 of 13 287 adolescents) in 2018, and 1.7% (242 of 13 397 adolescents) in 2019 (OR, 1.01; 95% CI, 0.99-1.04; *P* = .35). The Figure shows the annual prevalence of overall treatment use for MDE and SUD in the past year among adolescents with co-occurring conditions. In 2011, 100 of 280 adolescents (28.5%) with both conditions received treatment for MDE only, and the prevalence increased significantly to 102 of 242 adolescents (42.5%) in 2019 (OR, 1.07; 95% CI, 1.02-1.11; *P* = .005). In contrast, a decreasing trend was observed in adolescents’ treatment use for SUD only, from 8 of 280 adolescents (4.8%) in 2011 to 6 of 242 adolescents (1.5%) in 2019 (OR, 0.92; 95% CI, 0.85-0.99; *P* = .04).

**Figure.**
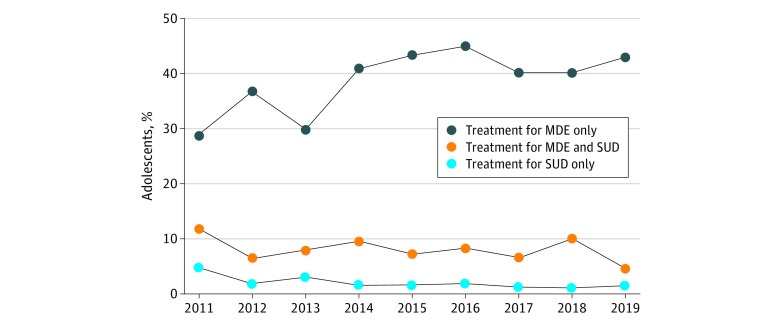
Patterns and Trends in Treatment Use Among Adolescents With Co-occurring Major Depressive Episode (MDE) and Substance Use Disorder (SUD) in the Past 12 Months in the National Survey on Drug Use and Health Weighted percentages are reported. Treatment categories are mutually exclusive.

From 2011 to 2019, there was no statistically significant linear trend in annual prevalence of treatment use for both MDE and SUD. In 2011, at its highest, prevalence was 11.6% (23 of 280 adolescents), and prevalence was lowest in 2019, at 4.5% (12 of 242 adolescents), (OR, 0.95; 95% CI, 0.87-1.03; *P* = .24).

Table 2 lists the annual prevalence of treatment use for MDE and SUD from different sources of service among adolescents with both conditions. Overall, the most common sources of treatment use for MDE included private therapists, school mental health professionals, mental health clinics, and in-home counselors. From 2011 to 2019, a significant increase was observed in adolescent treatment use for MDE from private therapists (OR, 1.07; 95% CI, 1.02-1.12; *P* = .003) and mental health clinics (OR, 1.08; 95% CI, 1.03-1.14; *P* = .003). In contrast, the most common sources of treatment use for SUD were mental health clinics, self-help groups, hospitals, and outpatient rehabilitation facilities. Across the survey years, a significant decrease was noted in SUD treatment use from outpatient rehabilitation facilities (OR, 0.86; 95% CI, 0.77-0.96; *P* = .007) and self-help groups (OR, 0.88; 95% CI, 0.80-0.97; *P* = .01).

**Table 2.** Patterns and Trends in the Prevalence of Treatment Use by Sources of Service for MDE and SUD Among Adolescents With Co-occurring MDE and SUD in the National Survey on Drug Use and Health

Source of service	Adolescents per survey year, % (95% CI)^a^									OR (95% CI)
	2011 (n = 280)	2012 (n = 280)	2013 (n = 258)	2014 (n = 188)	2015 (n = 205)	2016 (n = 192)	2017 (n = 213)	2018 (n = 214)	2019 (n = 242)	
**MDE treatment use^b^**										
Overnight in hospital	8.3 (4.3-12.3)	7.6 (4.1-11.1)	7.2 (3.7-10.7)	16.5 (9.5-23.4)	15.8 (9.2-22.3)	19.4 (12.5-26.3)	11.7 (6.6-16.9)	8.4 (3.7-13.1)	12.9 (7.1-18.7)	1.06 (0.99-1.12)
Residential treatment facility	4.1 (1.2-7.0)	6.7 (3.2-10.1)	5.1 (2.0-8.1)	10.3 (5.0-15.6)	8.6 (3.3-13.8)	13.9 (7.6-20.2)	6.7 (2.6-10.8)	5.2 (1.3-9.2)	10.9 (5.2-16.6)	1.07 (0.99-1.16)
Day treatment programs	6.6 (3.5-9.8)	8.5 (4.6-12.5)	4.6 (1.6-7.6)	9.1 (4.1-14.1)	10.9 (5.4-16.4)	14.8 (8.7-21.0)	8.7 (3.9-13.5)	8.7 (3.9-13.6)	7.8 (3.4-12.3)	1.04 (0.97-1.11)
Mental health clinic	12.1 (7.6-16.6)	12.7 (7.9-17.4)	10.7 (6.2-15.2)	16.3 (9.8-22.8)	27.1 (19.0-35.2)	28.1 (20.2-36.1)	19.2 (12.8-25.6)	14 (8.5-19.5)	21.5 (14.5-28.5)	1.08 (1.03-1.14)^c^
Private therapist	34.2 (26.7-41.7)	33.7 (26.5-40.9)	32.4 (24.9-40.0)	46.4 (37.3-55.4)	44.5 (35.5-53.5)	46.7 (37.9-55.6)	44 (35.5-52.5)	46.5 (36.9-56.1)	43.6 (35.2-52.0)	1.07 (1.02-1.12)^c^
In-home counselor	12.3 (7.9-16.8)	8.8 (4.9-12.7)	12.4 (6.7-18.1)	18.4 (11.2-25.6)	18.8 (11.3-26.3)	20.1 (13.1-27.0)	9.8 (4.9-14.6)	14.4 (6.9-21.9)	13 (7.2-18.9)	1.07 (1.02-1.11)
School social worker, counselor, or psychologist	23.5 (16.7-30.3)	25.5 (18.5-32.5)	21.2 (14.8-27.5)	27.4 (19.3-35.5)	26.8 (18.7-34.9)	33.2 (24.4-41.9)	29.5 (21.5-37.5)	31.6 (22.4-40.8)	27.9 (20.2-35.7)	1.05 (0.99-1.10)
Special school or program	12.3 (6.9-17.6)	16.1 (9.5-22.7)	10.8 (6.2-15.5)	11.1 (4.5-17.7)	14.3 (8.3-20.3)	19.1 (12.1-26.0)	12.1 (6.3-17.8)	17.7 (10.2-25.2)	14.4 (8.4-20.5)	1.03 (0.97-1.10)
Pediatrician or family physician	8.9 (4.1-13.8)	15.7 (9.9-21.6)	7.3 (3.9-10.6)	8.3 (4.0-12.6)	12.5 (6.6-18.5)	18.5 (11.0-26.0)	14.2 (7.8-20.6)	12.6 (6.1-19.1)	11.7 (6.2-17.2)	1.04 (0.97-1.11)
Juvenile detention, prison, or jail^d^	3.3	0.3	1.2	2.6	0	2.2	0.4	0.2	1.1	0.86 (0.73-1.02)
Foster care	1.3 (0.0-2.6)	2.5 (0.1-5.0)	0.5 (0.0-1.1)	0.9 (0.1-2.0)	2.1 (0.0-4.4)	2.7 (0.0-5.5)	1.3 (0.0-3.2)	0.9 (0.0-2.1)	0.6 (0.1-1.4)	0.94 (0.82-1.08)
**SUD treatment use^e^**										
Overnight in hospital	4.9 (1.1-8.8)	2.1 (0.2-4.0)	0.9 (0.3-2.2)	6.1 (1.3-10.9)	2.1 (0.0-4.2)	4.1 (0.2-8.0)	2.5 (0.4-4.7)	4.5 (1.2-7.7)	0.4 (0.0-1.2)	0.94 (0.84-1.05)
Residential rehabilitation facility	3.9 (0.8-7.0)	1.4 (0.0-3.1)	0.7 (0.0-1.8)	2.2 (0.0-4.5)	0.7 (0.0-1.7)	4.6 (0.7-8.7)	1.5 (0.0-3.0)	1.6 (0.0-3.3)	0.4 (0.0-1.2)	0.91 (0.79-1.05)
Outpatient rehabilitation facility	4.4 (1.2-7.6)	4.4 (1.0-7.8)	3.8 (0.2-7.4)	3.0 (0.6-5.4)	1.7 (0.0-3.5)	4.8 (0.9-8.8)	2.8 (0.5-5.1)	1.7 (0.0-3.6)	0.4 (0.0-1.0)	0.86 (0.77-0.96)^c^
Mental health clinic	6.1 (2.1-10.0)	3.6 (0.8-6.3)	3.0 (0.0-6.1)	2.8 (0.5-5.1)	2.9 (0.6-5.2)	6.1 (1.8-10.4)	2.5 (0.2-10.8)	3.9 (0.6-7.2)	1.6 (0.1-3.0)	0.93 (0.83-1.03)
Private physician’s office	1.8 (0.0-4.0)	1.6 (0.0-3.7)	0.6 (0.0-1.5)	4.3 (1.0-7.6)	1.1 (0.0-2.7)	1.5 (0.0-3.4)	2.1 (0.0-4.9)	4.3 (0.0-9.2)	0.6 (0.0-1.4)	1.02 (0.87-1.20)
Emergency department^d^	2.0	1.7	0.0	5.4	1.3	3.8	1.4	2.3	0.1	0.95 (0.85-1.06)
Prison or jail^d^	0.1	0.0	0.3	1.1	1.4	0.0	0.0	0.2	0.5	1.03 (0.84-1.26)
Self-help group^d^	3.7	3.4	4.6	4.8	3.3	3.4	3.6	2.2	0.0	0.88 (0.80-0.97)^f^

Abbreviations: OR, odds ratio; MDE, major depressive episode; SUD, substance use disorder.

^a^ Weighted percentages are presented. Specific sources of service use are not mutually exclusive. No mathematical correction was made for multiple comparisons.

^b^ All adolescents were asked if they had received treatment or counseling from each of 11 specialty and nonspecialty sources of service for behavioral or emotional problems that were not caused by alcohol or drug use. When restricting analysis to adolescents with co-occurring MDE and SUD, there may be overreport bias by adolescents who sought treatment for problems other than MDE or underreport bias by those whose emotional or behavioral problems were caused by alcohol or drug use. Therefore, these estimations are for illustration purpose and should be interpreted with caution.

^c^ *P* ≤ .01.

^d^ 95% CIs could not be computed for percentages owing to zero observed values.

^e^ Adolescents were asked if they had used treatment or counseling for illicit drug or alcohol use at any of 8 designated locations in the past 12 months.

^f^ *P* ≤ .05.

### Disparities in the Prevalence of Co-occurring MDE and SUD

As shown in Table 3, compared with boys and adolescents aged 12 to 13 years, higher prevalence of co-occurring conditions was observed in girls (AOR, 2.71; 95% CI, 2.37-3.10; *P* < .001) and adolescents aged 14 to 15 years (AOR, 6.11; 95% CI, 4.75-7.88; *P* < .001) and 16 to 17 years (AOR, 11.69; 95% CI, 9.17-14.90; *P* < .001). Meanwhile, compared with White adolescents, lower prevalence of co-occurring conditions was found in Black adolescents (AOR, 0.38; 95% CI, 0.30-0.49; *P* < .001) and Asian, Native Hawaiian, or Pacific Islander adolescents (AOR, 0.59; 95% CI, 0.41-0.86; *P* = .005). Consistently, having no father in the home (AOR,1.23; 95% CI, 1.07-1.42; *P* = .005) or no mother in the home (AOR, 1.38; 95% CI, 1.16-1.65; *P* < .001) were both associated with increased odds that adolescents had both conditions. Lastly, an overall higher prevalence of co-occurring conditions was found in adolescents who moved in the last year (Table 3).

**Table 3.** Multivariate Differences in the Prevalence of 12-Month Co-occurring MDE and SUD Among Adolescents in the National Survey on Drug Use and Health From 2011 to 2019

Characteristic	No. (N = 136 262)	Co-occurring MDE and SUD^a^	
		Prevalence, % (95% CI)	AOR (95% CI)
Year	NA	NA	1.01 (0.99-1.03)
Gender			
Boys	69 584	0.8 (0.7-0.9)	1 [Reference]
Girls	66 678	2.1 (2.0-2.3)	2.71 (2.37-3.10)^b^
Age, y			
12-13	43 547	0.2 (1.2-0.3)	1 [Reference]
14-15	46 167	1.4 (1.3-1.5)	6.11 (4.75-7.88)^b^
16-17	46 548	2.7 (2.5-2.9)	11.68 (9.17-14.90)^b^
Race and ethnicity			
Asian, Native Hawaiian, or Pacific Islander	5699	1.0 (0.6-1.3)	0.59 (0.41-0.86)^c^
Black	18 173	0.7 (0.6-0.9)	0.38 (0.30-0.49)^b^
Hispanic	28 687	1.5 (1.3-1.7)	0.94 (0.79-1.11)
White	74 512	1.6 (1.5-1.7)	1 [Reference]
Other^d^	9191	2.2 (1.7-2.6)	1.32 (1.04-1.68)^e^
Insurance type			
No	7859	1.4 (1.1-1.8)	1 [Reference]
Medicaid or CHIP	46 094	1.5 (1.3-1.6)	1.18 (0.90-1.56)
Private insurance	78 885	1.5 (1.3-1.6)	1.12 (0.86-1.46)
Other insurance	2003	1.5 (0.7-2.2)	1.01 (0.57-1.78)
Family income, $			
<20 000	23 261	1.3 (1.2-1.6)	1 [Reference]
20 000-49 999	40 112	1.6 (1.4-1.7)	1.17 (0.96-1.43)
50 000-74 999	21 942	1.3 (1.2-1.5)	1.06 (0.83-1.34)
≥75 000	50 947	1.5 (1.3-1.6)	1.16 (0.91-1.47)
Father in household			
Yes	98 170	1.4 (1.3-1.5)	1 [Reference]
No	37 968	1.7 (1.5-1.8)	1.23 (1.07-1.42)^c^
Mother in household			
Yes	124 542	1.4 (1.3-1.5)	1 [Reference]
No	11 605	2.2 (1.9-2.5)	1.38 (1.16-1.65)^b^
Times family moved in past 12 mo			
None	98 708	1.4 (1.3-1.5)	1 [Reference]
1	19 627	1.7 (1.4-1.9)	1.29 (1.09-1.52)^c^
2	9594	1.4 (1.1-1.7)	1.18 (0.94-1.50)
≥3	4956	2.8 (2.2-3.4)	2.18 (1.71-2.77)^b^

Abbreviations: AOR, multivariate adjusted odds ratio; CHIP, Children’s Health Insurance Program; MDE, major depressive episode; NA, not applicable; SUD, substance use disorders.

^a^ All variables listed were included in the multivariable model to estimate the prevalence of MDE, SUD, and co-occurring MDE and SUD. Unweighted sample sizes and weighted percentages are presented.

^b^ *P* ≤ .001.

^c^ *P* ≤ .01.

^d^ Includes individuals identifying as more than 1 race or ethnicity or as none of the provided race and ethnicity options.

^e^ *P* ≤ .05.

### Disparities in the Treatment of Co-occurring MDE and SUD

As listed in Table 4, among adolescents with co-occurring conditions, higher levels of treatment use were found in girls than in boys for MDE only (AOR, 2.00; 95% CI, 1.49-2.69; *P* < .001). Compared with adolescents aged 12 to 13 years, higher levels of treatment use were noted in adolescents aged 14 to 15 years and 16 to 17 years for SUD only and for co-occurring conditions. Compared with White adolescents, lower levels of treatment use were observed in Hispanic adolescents for co-occurring conditions (AOR, 0.52; 95% CI, 0.27-0.98; *P* = .04) and in Asian, Native Hawaiian, or Pacific Islander adolescents for MDE only (AOR, 0.24; 95% CI, 0.10-0.58; *P* < .001) and for co-occurring conditions (AOR, 0.04; 95% CI, 0.01-0.33; *P* = .003). Moving households 3 or more times in the past 12 months was associated with higher odds that adolescents received treatment for both conditions (AOR, 2.52; 95% CI, 1.26-5.05; *P* = 009). Having private insurance significantly increased the odds that adolescents used treatment for MDE only (AOR, 2.07; 95% CI, 1.09-3.91; *P* = .03). Lastly, having MDE-related severe impairment was associated with higher odds that adolescents received treatment for MDE only (AOR, 2.77; 95% CI, 1.97-3.90; *P* < .001) and for both conditions (AOR, 2.61; 95% CI, 1.30-5.23; *P* = .007).

**Table 4.** Multivariate Differences in the Prevalence of Treatment Use in Adolescents With Co-occurring MDE and SUD in the National Survey on Drug Use and Health From 2011 to 2019

Sociodemographic characteristics	No. (N = 2072)	Treatment for MDE only^a^		Treatment for SUD only^b^			Treatment for both MDE and SUD		
		% (95% CI)	AOR (95% CI)	% (95% CI)	AOR (95% CI)	% (95% CI)		AOR (95% CI)
Year		NA	1.06 (1.01-1.11)^c^	NA	0.85 (0.73-1.00)	NA		0.95 (0.87-1.04)
Gender								
Boys	548	26.5 (21.6-31.4)	1 [Reference]	2.6 (1.0-4.2)	1 [Reference]	9.8 (6.4-13.3)		1 [Reference]
Girls	1524	42.6 (39.3-46.0)	2.00 (1.49-2.69)^d^	1.8 (0.7-2.9)	0.81 (0.33-2.01)	7.2 (5.4-9.0)		0.62 (0.38-1.02)
Age								
12-13	123	44.2 (32.9-55.5)	1 [Reference]	0.0^e^	1 [Reference]	2.1 (0.0-4.3)		1 [Reference]
14-15	658	39 (34.1-43.9)	0.84 (0.51-1.39)	1.8^e^	NA	7.7 (4.9-10.5)		3.99 (1.18-13.41)^c^
16-17	1291	37.2 (33.7-40.8)	0.85 (0.52-1.38)	2.3^e^	NA	8.5 (6.4-10.7)		4.21 (1.31-13.45)^c^
Race and ethnicity								
Asian, Native Hawaiian, or Pacific Islander	56	14.8 (4.2-25.5)	0.24 (0.10-0.58)^d^	3.1 (0.0-9.0)	2.35 (0.26-21.3)	0.4 (0.0-1.3)		0.04 (0.01-0.33)^f^
Black	140	36.3 (25.5-47.1)	0.87 (0.51-1.51)	1.2 (0.0-2.8)	0.70 (0.14-3.46)	4.3 (0.0-8.7)		0.39 (0.14-1.13)
Hispanic	463	34.9 (28.7-41.1)	0.84 (0.60-1.19)	3.0 (0.2-5.8)	1.72 (0.59-5.02)	5.4 (2.8-7.9)		0.52 (0.27-0.98)^c^
White	1224	40.8 (37.3-44.3)	1 [Reference]	1.5 (0.6-2.4)	1 [Reference]	9.6 (7.2-11.9)		1 [Reference]
Other^g^	189	41.6 (31.4-51.9)	1.05 (0.65-1.67)	3.6 (0.0-7.2)	2.05 (0.55-7.66)	10.8 (2.8-18.8)		1.05 (0.46-2.35)
Insurance type								
No	123	24.6 (13.9-35.2)	1 [Reference]	2.8 (0.0-6.9)	1 [Reference]	4 (0.5-7.4)		1 [Reference]
Medicaid or CHIP	724	36.7 (31.8-41.5)	1.90 (0.98-3.68)	3.1 (1.0-5.3)	1.14 (0.24-5.32)	9 (6.0-11.9)		2.36 (0.88-6.30)
Private insurance	1164	40.7 (37.0-44.4)	2.07 (1.09-3.91)^c^	1.3 (0.4-2.1)	0.40 (0.08-2.15)	7.9 (5.7-10.1)		2.28 (0.82-6.30)
Other insurance	33	28.4 (7.0-49.9)	1.27 (0.39-4.16)	2.2 (0.0-6.5)	0.71 (0.06-8.99)	3.5 (0.0-8.5)		0.78 (0.13-4.88)
Family income, $								
<20 000	334	29.2 (22.3-36.0)	1 [Reference]	2.7 (0.5-4.9)	1 [Reference]	10.3 (5.7-14.9)		1 [Reference]
20 000-49 999	675	39 (34.0-44.0)	1.42 (0.92-2.19)	2.2 (0.6-3.8)	0.81 (0.26-2.47)	7.7 (5.0-10.3)		0.79 (0.42-1.49)
50 000-74 999	325	36.9 (30.1-43.7)	1.16 (0.69-1.93)	3.0 (0.0-7.3)	1.13 (0.21-5.90)	4.4 (1.8-7.0)		0.44 (0.19-1.00)
≥75 000	738	41.4 (36.8-46.0)	1.38 (0.84-2.26)	1.3 (0.4-2.2)	0.46 (0.15-1.41)	8.5 (5.6-11.3)		0.90 (0.43-1.86)
Father in household								
Yes	1396	38.8 (35.4-42.2)	1 [Reference]	1.9 (0.8-3.1)	1 [Reference]	7.3 (5.4-9.2)		1 [Reference]
No	675	36.8 (32.0-41.6)	1.01 (0.75-1.35)	2.3 (0.8-3.7)	1.19 (0.54-2.66)	9.4 (6.4-12.4)		1.22 (0.77-1.93)
Mother in household								
Yes	1805	37.6 (34.6-40.6)	1 [Reference]	2.0 (1.0-3.0)	1 [Reference]	7.6 (5.9-9.3)		1 [Reference]
No	266	42.4 (34.9-49.9)	1.24 (0.88-1.75)	2.3 (0.0-4.7)	1.23 (0.41-3.73)	10.3 (5.7-14.9)		1.15 (0.64-2.09)
Times family moved in past 12 mo								
None	1395	38.5 (35.1-41.9)	1 [Reference]	2.5 (1.2-3.7)	1 [Reference]	6.8 (5.0-8.6)		1 [Reference]
1	350	38.5 (31.6-45.4)	0.92 (0.65-1.31)	1.1 (0.0-2.4)	0.45 (0.13-1.56)	10.3 (5.4-15.2)		1.65 (0.91-3.00)
2	141	34.7 (24.5-45.0)	0.78 (0.49-1.24)	1.6 (0.0-3.9)	0.67 (0.15-3.02)	6.9 (1.9-11.9)		1.05 (0.46-2.43)
≥3	157	34.6 (24.4-44.8)	0.94 (0.57-1.53)	0.5 (0.0-1.5)	0.12 (0.12-1.04)	16 (8.0-24.0)		2.52 (1.26-5.05)^f^
MDE-related severe impairment								
No	383	21.7 (16.4-26.9)	1 [Reference]	4.0 (0.3-7.7)	1 [Reference]	3.6 (1.4-5.8)		1 [Reference]
Yes	1684	42.3 (39.2-45.5)	2.77 (1.97-3.90)^d^	1.5 (0.9-2.2)	0.45 (0.18-1.14)	9 (7.1-10.9)		2.61 (1.30-5.23)^f^

Abbreviations: AOR, multivariate adjusted odds ratio; CHIP, Children’s Health Insurance Program; MDE, major depressive episode; NA, not applicable; SUD, substance use disorders.

^a^ Adolescents were asked if they had seen or talked to a physician or other professionals about their MDE symptoms in the past 12 months.

^b^ Adolescents were asked if they had used treatment or counseling for illicit drug use or alcohol use at any location designed in the past 12 months.

^c^ *P* ≤ .05.

^d^ *P* ≤ .001.

^e^ 95% CIs could not be computed for percentages owing to zero observed values.

^f^ *P* ≤ .01.

^g^ Includes individuals identifying as more than 1 race or ethnicity or as none of the provided race and ethnicity options.

## Discussion

This survey study found that from 2011 to 2019, the annual prevalence of co-occurring MDE and SUD remained largely stable, at between 1.4% to 1.7%, among US adolescents. Each year, less than 12% of adolescents with co-occurring MDE and SUD received treatment for both conditions, without a significant linear trend over time. In the past decade, multiple policies have been implemented to expand mental health and substance use treatment for adolescents, including the Mental Health Parity and Addiction Equity Act^21^ and the Patient Protection and Affordable Care Act (ACA).^22^ Nevertheless, persistent treatment gaps were noted. Currently, there are approximately 8300 practicing child and adolescent psychiatrists nationwide, with more than 15 million children and adolescents in need of specialty services.^23,24^ In 2018, 75% of counties in the US had no child psychiatrist, and 20% of medical schools did not sponsor child and adolescent psychiatry residency programs.^25^ To meet adolescents’ treatment needs for MDE and SUD, more funding and policy support are needed to address such workforce shortages and deficiencies in treatment infrastructure.

Overall, adolescents with both conditions received treatment for MDE at much higher levels than for SUD, and a significant decrease in SUD treatment use was observed over time, particularly in outpatient rehabilitation facilities and self-help groups. Historically, SUD treatment in the US has not been well-integrated into the mental health care system and is often provided by multiple systems.^26,27,28^ To improve adolescent treatment use for SUD, enhanced service coordination between health care systems is essential. Currently, more than 70% of SUD treatment facilities in the US do not have adolescent-specific programs, and adolescents with SUD service needs are often integrated into programs that serve adults.^29,30^ To ensure adolescents receive adequate treatment for SUD, more adolescent-specific programs that recognize the unique developmental characteristics and emotional needs of adolescents are needed. The difference in the prevalence of MDE and SUD treatment may also be explained by the greater stigma associated with SUD than MDE.^31^ More community and school outreach programs are necessary to improve treatment motivation for SUD in adolescents.

Disaggregated analysis of treatment use by sources of service indicates that most adolescents with co-occurring conditions received specialty services from therapists, mental health clinics, and counselors for their MDE. In comparison, a much smaller proportion of adolescents received SUD treatment in mental health clinics, suggesting that either adolescents were underdiagnosed for their SUD, or they were receiving segregated rather than integrated care for their co-occurring conditions. Compared with separate treatment for each condition, integrated care has superior quality, effectiveness, and efficiency in treating co-occurring mental health and substance use problems.^32,33^ To optimize treatment outcomes for adolescents, enhanced efforts are needed to increase the provision of integrated care in clinical settings.

Another strategy would be to incorporate MDE and SUD services into primary care, which could reach far more adolescents through routine checkups.^34^ In recent years, many efforts have been made to improve mental health and substance use management in pediatric settings, including the guidelines for adolescent depression in primary care,^35^ the Screening, Brief Intervention, and Referral to Treatment (SBIRT) for SUD,^36^ and the patient-centered medical home model under the ACA.^37^ Nevertheless, this survey study found no significant change in adolescent treatment use for MDE or SUD from family physicians or pediatricians over time. A recent survey with pediatricians nationwide found that only 26% of pediatricians used validated screening instruments for substance use in clinical practice, 11% implemented the full SBIRT model, and 68% made referrals to substance use specialty care in response to a positive screen.^38^ To enhance implementation of evidence-based interventions, such as SBIRT, in pediatric settings, barriers perceived by pediatricians, including confidentiality issues, insufficient time during appointments, lack of expertise for managing substance use, and limited access to referral services, need to be addressed.^38^

This study also found extensive disparities in adolescent unmet treatment needs for co-occurring MDE and SUD. For example, while a higher level of treatment use were observed in girls than in boys for MDE, no gender difference was found in treatment use for SUD or co-occurring conditions. Such underutilization of MDE treatment in boys and insufficient service use for SUD in girls may be explained by gender stereotypes regarding proneness to emotional problems in girls and substance use problems in boys, which may create barriers to accurate identification and treatment of both disorders.^39,40^ Furthermore, although experiencing mental health problems is socially undesirable in general, the perception of stigma and shame is especially strong for adolescent boys.^41,42^ Taken together, these findings point to the need for gender-sensitive diagnosis and gender-responsive efforts to engage adolescents into treatment.

Additionally, compared with adolescents aged 12 to 13 years, higher prevalence of co-occurring conditions was noted in those aged 14 to 15 years and 16 to 17 years. Although corresponding higher treatment use was found for SUD and co-occurring conditions among older adolescents, there was no age difference in MDE treatment, highlighting the need for enhanced MDE management in high schools. As reported in this study, a large proportion of adolescents received school-based services for their MDE. Because of limited resources and budget restrictions, high schools often have difficulty hiring enough mental health professionals to handle heavy caseloads.^43^ To ensure adolescents receive timely and accessible treatment for MDE, strengthened funding support is needed to integrate the community mental health workforce into the school system.

Notably, lower prevalence of treatment use for co-occurring conditions was found in Hispanic adolescents than in White adolescents. Additionally, among all racial and ethnic groups, Asian American, Native Hawaiian, and Pacific Islander adolescents had the lowest prevalence of treatment use for MDE and both conditions. Previous research has identified multiple culturally unique barriers that may prevent racial and ethnic minority adolescents, such as Asian American, Native Hawaiian, and Pacific Islander adolescents, from seeking mental health treatment, including language barriers, cultural mistrust of health care practitioners, lack of social support, and limited provision of services in the community.^44,45^ To improve treatment use for depression and SUD among racial and ethnic minority adolescents, more culturally acceptable and accessible interventions are crucial.

Moreover, we found that having private insurance was associated with treatment use for MDE, but not for SUD or co-occurring conditions. This finding is largely consistent with previous research identifying positive associations of insurance coverage with adolescent mental health treatment,^46,47,48^ but not with SUD treatment.^49,50^ Mental health and SUD treatment have been designated as essential health benefits under the ACA.^51^ With further implementation of ACA and increased awareness of the policy, it is anticipated that more adolescents with co-occurring MDE and SUD who are covered by Medicaid will receive the treatment they need.

Lastly, although findings from this study identified higher odds of co-occurring MDE and SUD and unmet treatment needs among adolescents in single-parent households and those whose families who moved households frequently, further research is needed to clarify potential associations among socioeconomic status, family structure, and residential instability. As single parenthood and residential instability are largely markers associated with underlying social, educational, and familial disadvantages,^18,19^ mental health and SUD interventions should target more on providing social support for single-parent households and improving family functioning.

### Limitations

This study has several limitations. First, the NSDUH was administered in English and Spanish only, which overlooked immigrant families of other racial and ethnic groups with limited English proficiency. Second, the NSDUH survey excludes people (including adolescents) experiencing homelessness who do not use shelters and residents of hospitals and institutional group quarters (eg, juvenile centers); these individuals may have higher treatment needs for MDE and SUD. Third, the small sample sizes of adolescents with co-occurring MDE and SUD in each year and by specific sociodemographic characteristics may not have the statistical power to detect any small difference, possibly resulting in type II errors.^52^ Fourth, the small sample sizes of adolescents who used treatment in specific settings restricted our capability to conduct any setting-specific examination. Future studies with larger sample sizes are needed to explore disparities and factors associated with adolescent MDE and SUD treatment use in specific settings.

## Conclusions

This survey study found a stable trend in the prevalence and persistent gap in the treatment of co-occurring MDE and SUD among adolescents in the US from 2011 to 2019. Findings from this study point to ongoing deficiencies in the current service capacity for adolescent mental health and SUD treatment, highlight the need for improved coordination between service delivery systems, and call for enhanced policy and funding support for adolescents with unmet treatment needs.

## References

[1] Jordan CJ, Andersen SL. Sensitive periods of substance abuse: early risk for the transition to dependence. Dev Cogn Neurosci. 2017;25:29-44. doi:10.1016/j.dcn.2016.10.004 27840157 PMC5410194

[2] Chambers RA, Taylor JR, Potenza MN. Developmental neurocircuitry of motivation in adolescence: a critical period of addiction vulnerability. Am J Psychiatry. 2003;160(6):1041-1052. doi:10.1176/appi.ajp.160.6.1041 12777258 PMC2919168

[3] Casey BJ, Jones RM. Neurobiology of the adolescent brain and behavior: implications for substance use disorders. J Am Acad Child Adolesc Psychiatry. 2010;49(12):1189-1201. doi:10.1097/00004583-201012000-00005 21093769 PMC3099425

[4] US Department of Health and Human Services. Report to Congress on the Prevention and Treatment of Co-occurring Substance Abuse and Mental Disorders. Substance Abuse and Mental Health Services Administration; 2002.

[5] Grella CE, Hser YI, Joshi V, Rounds-Bryant J. Drug treatment outcomes for adolescents with comorbid mental and substance use disorders. J Nerv Ment Dis. 2001;189(6):384-392. doi:10.1097/00005053-200106000-00006 11434639

[6] Kandel DB, Johnson JG, Bird HR, . Psychiatric disorders associated with substance use among children and adolescents: findings from the Methods for the Epidemiology of Child and Adolescent Mental Disorders (MECA) Study. J Abnorm Child Psychol. 1997;25(2):121-132. doi:10.1023/A:1025779412167 9109029

[7] Grucza RA, Krueger RF, Agrawal A, . Declines in prevalence of adolescent substance use disorders and delinquent behaviors in the USA: a unitary trend? Psychol Med. 2018;48(9):1494-1503. doi:10.1017/S0033291717002999 29065935 PMC6217939

[8] Keyes KM, Hamilton A, Patrick ME, Schulenberg J. Diverging trends in the relationship between binge drinking and depressive symptoms among adolescents in the US from 1991 through 2018. J Adolesc Health. 2020;66(5):529-535. doi:10.1016/j.jadohealth.2019.08.026 31676228 PMC7183904

[9] Levy S, Ziemnik RE, Harris SK, . Screening adolescents for alcohol use: tracking practice trends of Massachusetts pediatricians. J Addict Med. 2017;11(6):427-434. doi:10.1097/ADM.0000000000000340 28731863

[10] Bukstein OG, Bernet W, Arnold V, ; Work Group on Quality Issues. Practice parameter for the assessment and treatment of children and adolescents with substance use disorders. J Am Acad Child Adolesc Psychiatry. 2005;44(6):609-621. doi:10.1097/01.chi.0000159135.33706.37 15908844

[11] Hinckley JD, Riggs P. Integrated treatment of adolescents with co-occurring depression and substance use disorder. Child Adolesc Psychiatr Clin N Am. 2019;28(3):461-472. doi:10.1016/j.chc.2019.02.006 31076120

[12] Schmitz JM, Averill P, Stotts AL, Moeller FG, Rhoades HM, Grabowski J. Fluoxetine treatment of cocaine-dependent patients with major depressive disorder. Drug Alcohol Depend. 2001;63(3):207-214. doi:10.1016/S0376-8716(00)00208-8 11418225

[13] Riggs PD, Baker S, Mikulich SK, Young SE, Crowley TJ. Depression in substance-dependent delinquents. J Am Acad Child Adolesc Psychiatry. 1995;34(6):764-771. doi:10.1097/00004583-199506000-00017 7608050

[14] Lu W. Adolescent depression: national trends, risk factors, and healthcare disparities. Am J Health Behav. 2019;43(1):181-194. doi:10.5993/AJHB.43.1.15 30522576

[15] Substance Abuse and Mental Health Services Administration. National Survey on Drug Use and Health. Accessed January 1, 2019. https://www.samhsa.gov/data/data-we-collect/nsduh-national-survey-drug-use-and-health

[16] American Psychiatric Association. Diagnostic and Statistical Manual of Mental Disorders. 4th ed. American Psychiatric Association; 1994.

[17] Mojtabai R, Olfson M, Han B. National trends in the prevalence and treatment of depression in adolescents and young adults. Pediatrics. 2016;138(6):e20161878. doi:10.1542/peds.2016-1878 27940701 PMC5127071

[18] Fergusson DM, Boden JM, Horwood LJ. Exposure to single parenthood in childhood and later mental health, educational, economic, and criminal behavior outcomes. Arch Gen Psychiatry. 2007;64(9):1089-1095. doi:10.1001/archpsyc.64.9.1089 17768274

[19] Jelleyman T, Spencer N. Residential mobility in childhood and health outcomes: a systematic review. J Epidemiol Community Health. 2008;62(7):584-592. doi:10.1136/jech.2007.060103 18559440

[20] Dong Y, Peng CY. Principled missing data methods for researchers. Springerplus. 2013;2(1):222. doi:10.1186/2193-1801-2-222 23853744 PMC3701793

[21] Mulvaney-Day N, Gibbons BJ, Alikhan S, Karakus M. Mental Health Parity and Addiction Equity Act and the use of outpatient behavioral health services in the United States, 2005-2016. Am J Public Health. 2019;109(S3):S190-S196. doi:10.2105/AJPH.2019.305023 31242013 PMC6595520

[22] Patient Protection and Affordable Care Act, Pub L No. 111-148, 124 Stat 119 (2010). Accessed August 1, 2021. https://www.govinfo.gov/app/details/PLAW-111publ148

[23] American Academy of Child & Adolescent Psychiatry. Suicide in children and teens. Accessed July 12, 2021. https://www.aacap.org/ AACAP/Families_and_Youth/Facts_for_Families/ FFF-Guide/Teen-Suicide-010.aspx

[24] Hunt J, Reichenberg J, Lewis AL, Jacobson S. Child and adolescent psychiatry training in the USA: current pathways. Eur Child Adolesc Psychiatry. 2020;29(1):63-69. doi:10.1007/s00787-019-01402-9 31515613

[25] McBain RK, Kofner A, Stein BD, Cantor JH, Vogt WB, Yu H. Growth and distribution of child psychiatrists in the United States: 2007–2016. Pediatrics. 2019;144(6):e20191576. doi:10.1542/peds.2019-1576 31685696 PMC6889947

[26] Knudsen HK. Adolescent-only substance abuse treatment: availability and adoption of components of quality. J Subst Abuse Treat. 2009;36(2):195-204. doi:10.1016/j.jsat.2008.06.002 19000942 PMC2692409

[27] Cummings JR, Wen H, Ko M. Decline in public substance use disorder treatment centers most serious in counties with high shares of Black residents. Health Aff (Millwood). 2016;35(6):1036-1044. doi:10.1377/hlthaff.2015.1630 27269020 PMC5019357

[28] Alinsky RH, Hadland SE, Matson PA, Cerda M, Saloner B. Adolescent-serving addiction treatment facilities in the United States and the availability of medications for opioid use disorder. J Adolesc Health. 2020;67(4):542-549. doi:10.1016/j.jadohealth.2020.03.005 32336560 PMC7508760

[29] Asarnow JR, Rozenman M, Wiblin J, Zeltzer L. Integrated medical-behavioral care compared with usual primary care for child and adolescent behavioral health: a meta-analysis. JAMA Pediatr. 2015;169(10):929-937. doi:10.1001/jamapediatrics.2015.1141 26259143

[30] Corrigan PW, Lurie BD, Goldman HH, Slopen N, Medasani K, Phelan S. How adolescents perceive the stigma of mental illness and alcohol abuse. Psychiatr Serv. 2005;56(5):544-550. doi:10.1176/appi.ps.56.5.544 15872162

[31] Mark TL, Song X, Vandivort R, . Characterizing substance abuse programs that treat adolescents. J Subst Abuse Treat. 2006;31(1):59-65. doi:10.1016/j.jsat.2006.03.017 16814011

[32] Kelly TM, Daley DC. Integrated treatment of substance use and psychiatric disorders. Soc Work Public Health. 2013;28(3-4):388-406. doi:10.1080/19371918.2013.774673 23731427 PMC3753025

[33] Mueser KT, Gingerich S. Treatment of co-occurring psychotic and substance use disorders. Soc Work Public Health. 2013;28(3-4):424-439. doi:10.1080/19371918.2013.774676 23731429

[34] Knapp PK, Foy JM. Integrating mental health care into pediatric primary care settings. J Am Acad Child Adolesc Psychiatry. 2012;51(10):982-984. doi:10.1016/j.jaac.2012.07.009 23021473

[35] Zuckerbrot RA, Cheung A, Jensen PS, Stein REK, Laraque D; GLAD-PC Steering Group. Guidelines for Adolescent Depression in Primary Care (GLAD-PC): part I—practice preparation, identification, assessment, and initial management. Pediatrics. 2018;141(3):e20174081. doi:10.1542/peds.2017-4081 29483200

[36] Levy SJ, Williams JF; Committee on Substance Use and Prevention. Substance use screening, brief intervention, and referral to treatment. Pediatrics. 2016;138(1):e20161211. doi:10.1542/peds.2016-1211 27325634

[37] Richardson LP, McCarty CA, Radovic A, Suleiman AB. Research in the integration of behavioral health for adolescents and young adults in primary care settings: a systematic review. J Adolesc Health. 2017;60(3):261-269. doi:10.1016/j.jadohealth.2016.11.013 28087267 PMC5973784

[38] Hammond CJ, Parhami I, Young AS, . Provider and practice characteristics and perceived barriers associated with different levels of adolescent SBIRT implementation among a national sample of US pediatricians. Clin Pediatr (Phila). 2021;60(9-10):418-426. doi:10.1177/00099228211034334 34342242 PMC13065241

[39] Call JB, Shafer K. Gendered manifestations of depression and help seeking among men. Am J Mens Health. 2018;12(1):41-51. doi:10.1177/1557988315623993 26721265 PMC5734537

[40] Kloos A, Weller RA, Chan R, Weller EB. Gender differences in adolescent substance abuse. Curr Psychiatry Rep. 2009;11(2):120-126. doi:10.1007/s11920-009-0019-8 19302765

[41] Chandra A, Minkovitz CS. Stigma starts early: gender differences in teen willingness to use mental health services. J Adolesc Health. 2006;38(6):754.e1-754.e8. doi:10.1016/j.jadohealth.2005.08.011 16730608

[42] Rice SM, Telford NR, Rickwood DJ, Parker AG. Young men’s access to community-based mental health care: qualitative analysis of barriers and facilitators. J Ment Health. 2018;27(1):59-65. doi:10.1080/09638237.2016.1276528 28132568

[43] Ringeisen H, Miller S, Munoz B, Rohloff H, Hedden SL, Colpe LJ. Mental health service use in adolescence: findings from the National Survey on Drug Use and Health. Psychiatr Serv. 2016;67(7):787-789. doi:10.1176/appi.ps.201400196 27032654 PMC5951613

[44] Lu W, Todhunter-Reid A, Mitsdarffer ML, Muñoz-Laboy M, Yoon AS, Xu L. Barriers and facilitators for mental health service use among racial/ethnic minority adolescents: a systematic review of literature. Front Public Health. 2021;9:641605. doi:10.3389/fpubh.2021.641605 33763401 PMC7982679

[45] Falgas I, Ramos Z, Herrera L, . Barriers to and correlates of retention in behavioral health treatment among Latinos in two different host countries: US and Spain. J Public Health Manag Pract. 2017;23(1):e20-e27. doi:10.1097/PHH.0000000000000391 26910867 PMC5320890

[46] Burns BJ, Costello EJ, Erkanli A, Tweed DL, Farmer EMZ, Angold A. Insurance coverage and mental health service use by adolescents with serious emotional disturbance. J Child Fam Stud. 1997;6:89-111. doi:10.1023/A:1025024808146

[47] Cummings JR, Druss BG. Racial/ethnic differences in mental health service use among adolescents with major depression. J Am Acad Child Adolesc Psychiatry. 2011;50(2):160-170. doi:10.1016/j.jaac.2010.11.004 21241953 PMC3057444

[48] Kataoka SH, Zhang L, Wells KB. Unmet need for mental health care among U.S. children: variation by ethnicity and insurance status. Am J Psychiatry. 2002;159(9):1548-1555. doi:10.1176/appi.ajp.159.9.1548 12202276

[49] Wu P, Hoven CW, Tiet Q, Kovalenko P, Wicks J. Factors associated with adolescent utilization of alcohol treatment services. Am J Drug Alcohol Abuse. 2002;28(2):353-369. doi:10.1081/ADA-120002978 12014820 PMC3070427

[50] Wu P, Hoven CW, Fuller CJ. Factors associated with adolescents receiving drug treatment: findings from the National Household Survey on Drug Abuse. J Behav Health Serv Res. 2003;30(2):190-201. doi:10.1007/BF02289807 12710372 PMC3089901

[51] Ader J, Stille CJ, Keller D, Miller BF, Barr MS, Perrin JM. The medical home and integrated behavioral health: advancing the policy agenda. Pediatrics. 2015;135(5):909-917. doi:10.1542/peds.2014-3941 25869375

[52] Hoenig JM, Heisey DM. The abuse of power: the pervasive fallacy of power calculations for data analysis. Am Stat. 2001;55(1):19-24. doi:10.1198/000313001300339897

